# Impact of cuff-assisted colonoscopy for adenoma detection: A protocol of systematic review and meta-analysis

**DOI:** 10.1097/MD.0000000000020243

**Published:** 2020-05-15

**Authors:** Qi Li, Hai-de Gao, Chun-Cheng Liu, Hao Zhang, Xun-Hai Li, Jia Wu, Xian-Kai Zhang

**Affiliations:** a Department of Endoscopy Center, Dashiqiao Central Hospital, Dashiqiao; b Department of Digestive Surgery, The Medical Group of Zhengzhou First People's Hospital, Zhengzhou, 450004, China.

**Keywords:** adenoma detection, cuff-assisted colonoscopy, impact

## Abstract

**Background:**

Previous studies have reported that cuff-assisted colonoscopy (CAC) can be used for detection of adenoma (DA). However, there are inconsistent results regarding the CAC for DA. Thus, this study will systematically explore the impact of CAC for DA.

**Methods:**

In order to retrieve potential eligible articles, this study will identify the following electronic databases from their inceptions to present: MEDLINE, EMBASE, Cochrane Library, PSYCINFO, Web of Science, Chinese Biomedical Literature Database, and China National Knowledge Infrastructure. All electronic databases will be searched without any language limitation. We will consider case-controlled studies that focused on exploring the impacts of CAC for DA. Two authors will perform study selection, information collection and risk of bias assessment, respectively. Any discrepancies between 2 authors will be resolved through discussion with a third author.

**Results:**

This study will summarize the most recent evidence to assess the impact of CAC for DA.

**Conclusion:**

The findings of this study will provide evidence of CAC for DA in clinical practice.

**Systematic review registration:**

INPLASY202040042.

## 1 Introduction

Colorectal cancer is one of the most common cancers,^[1,2,3,4,5]^ which is also the leading cause of death around the world.^[6,7]^ Previous study has reported that adenocarcinoma accounts for more than 95% of malignant tumors.^[8,9]^ Thus, it is very important to detect adenoma at early stage.^[10,11,12]^ Detection of adenoma (DA) rate is an essential quality indicator during colonoscopy,^[13,14,15,16]^ which is also associated with colorectal cancer incidence and subsequent death.^[17]^

A variety of studies have found that cuff-assisted colonoscopy (CAC) can be used for DA.^[18,19,20]^ However, no systematic review has been conducted to check the impact of CAC for DA. Therefore, this study will investigate the impact of CAC for DA.

## 2 Methods

### 2.1 Study registration

We have registered this study on INPLASY202040042, and we report it according to the Preferred Reporting Items for Systematic Reviews and Meta-Analysis (PRISMA) Protocol statement guidelines.^[21]^

### 2.2 Eligibility criteria

#### 2.2.1 Type of studies

We will include case-controlled studies (CCSs) reporting the impacts of CAC for DA. All experimental studies, case studies, non-clinical studies, and non-controlled studies will be excluded.

#### 2.2.2 Type of participants

Any patients who were diagnosed with histological-proven adenoma will be included in this study without restrictions of race, age, sex, and country.

#### 2.2.3 Type of indexes

Experimental group: All participants received CAC for DA.

Control group: All participants underwent detection of histological-proven adenoma, but not CAC.

#### 2.2.4 Type of outcome measurements

The primary outcome measurements are sensitivity and specificity. The secondary outcome measurements are diagnostic odds ratio, adenoma detection rate, the number of diagnosed adenomas, polyp detection rate, and cecal intubation rate.

### 2.3 Data sources and search strategy

#### 2.3.1 Electronic searches

The following electronic databases will be searched in MEDLINE, EMBASE, Cochrane Library, PSYCINFO, Web of Science, Chinese Biomedical Literature Database, and China National Knowledge Infrastructure from their inceptions to the present. We will not apply any language and publication status limitations to the above electronic databases. All CCSs that focused on exploring the impacts of CAC for DA will be considered. A search strategy has been developed for MEDLINE (Table 1). We will also amend similar strategies for use in other databases.

**Table 1:**
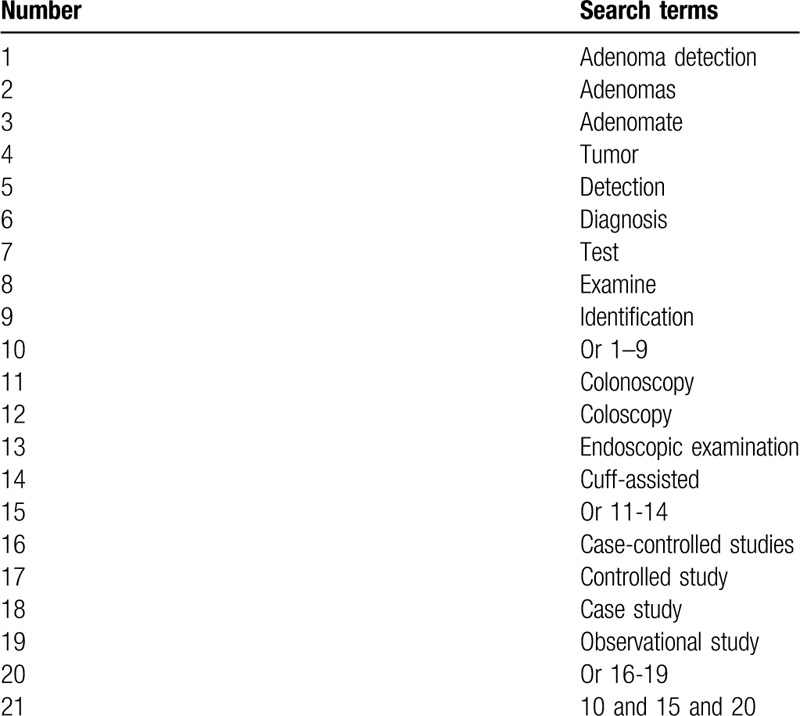
Detailed search strategy of MEDLINE.

#### 2.3.2 Other resources

We will also search grey records, such as dissertations, conference abstracts, and reference list of relevant reviews.

### 2.4 Data collection and analysis

#### 2.4.1 Selection of studies

All searched records will be entered into Endnote 7.0 software, and all duplicated studies will be removed automatically and manually. Two authors will independently check titles and abstracts of all retrieved literatures to evaluate eligibility for inclusion. After initial selection, full papers of potential studies will be further obtained to check eligibility for inclusion. The process of study identification will be presented in a flowchart. Any different opinions between 2 authors will be solved through consultation with the help of a third author.

#### 2.4.2 Data extraction

Two authors will independently extract data from each included study using predefined data collection sheet. The extracted information includes first author, publication time, study characteristics, patient characteristics, study design, study setting, study methods, details of indexes, outcome measurements, and any other relevant information. Any disagreements will be solved by a third author through discussion. If relevant essential information cannot be retrieved from the included articles, primary authors will be contacted to request it.

### 2.5 Study quality assessment

To determine the methodological quality for the included studies, Quality Assessment of Diagnostic Accuracy Studies tool^[22]^ will be used for CCSs. Two authors will independently evaluate the methodological quality for all included study. Any disagreements regarding study quality assessment between two authors will be resolved by consultation with a third author.

### 2.6 Statistical analysis

RevMan V.5.3 software will be used for data analysis in this study. All outcome data will be calculated as descriptive statistics or risk ratio and 95% confidence intervals. Whenever necessary, we will also perform a descriptive forest plot and a summary receiver operating characteristic. The degree of heterogeneity across eligible studies will be identified using *I*^*2*^ statistic. *I*^*2*^ ≤ 50% means low heterogeneity, while *I*^*2*^ > 50% means significant heterogeneity. If there is low heterogeneity, we will use a fixed-effects model and will carry out meta-analysis. If there is significant heterogeneity, we will use a random-effect model, and will perform subgroup analysis. If we can still detect substantial heterogeneity after subgroup analysis, we will conduct narrative summary to synthesize outcome data.

### 2.7 Subgroup analysis

We will perform subgroup analysis based on the different characteristics of study and patient, index types, and outcomes.

### 2.8 Sensitivity analysis

We will carry out sensitivity analysis to check robustness of pooled results by removing low quality studies.

### 2.9 Reporting bias

We will perform funnel plots to check any potential reporting bias when more than 10 studies are included.^[23]^

### 2.10 Ethics and dissemination

This study does not need formal ethical assessment or informed consent, because it will not analyze individual patient data. The findings of this study will be published on a peer-reviewed journal.

## 3 Discussion

Previous studies have reported that CAC can be used for DA. However, no systematic review has been conducted to test the impact of CAC for DA. This systematic review will firstly and systematically examine the impact of CAC for DA by evaluating sensitivity, specificity, diagnostic odds ratio, adenoma detection rate, the number of diagnosed adenomas, polyp detection rate, and cecal intubation rate. The results of this study may present a summary of the most recent evidence of CAC for DA, which may provide recommendation for both clinicians and future associated studies.

## Author contributions

**Conceptualization:** Qi Li, Hai-de Gao, Chun-cheng Liu.

**Data curation:** Qi Li, Hai-de Gao, Hao Zhang, Xun-hai Li, Jia Wu, Xian-kai Zhang.

**Formal analysis:** Qi Li, Chun-cheng Liu, Xun-hai Li, Jia Wu, Xian-kai Zhang.

**Funding acquisition:** Hai-de Gao.

**Investigation:** Hai-de Gao.

**Methodology:** Qi Li, Chun-cheng Liu, Hao Zhang, Xun-hai Li, Jia Wu.

**Project administration:** Hai-de Gao.

**Resources:** Qi Li, Chun-cheng Liu, Hao Zhang, Xun-hai Li, Jia Wu, Xian-kai Zhang.

**Software:** Qi Li, Chun-cheng Liu, Hao Zhang, Xun-hai Li, Jia Wu, Xian-kai Zhang.

**Supervision:** Hai-de Gao.

**Validation:** Qi Li, Hai-de Gao, Chun-cheng Liu, Xian-kai Zhang.

**Visualization:** Qi Li, Hai-de Gao, Hao Zhang, Xun-hai Li, Jia Wu, Xian-kai Zhang.

**Writing – original draft:** Qi Li, Hai-de Gao, Chun-cheng Liu, Xun-hai Li, Xian-kai Zhang.

**Writing – review & editing:** Qi Li, Hai-de Gao, Hao Zhang, Jia Wu.

## References

[1] Mattiuzzi C Sanchis-Gomar F Lippi G Concise update on colorectal cancer epidemiology. Ann Transl Med 2019;7:609. 32047770 10.21037/atm.2019.07.91 PMC7011596

[2] Chong RC Ong MW Tan KY Managing elderly with colorectal cancer. J Gastrointest Oncol 2019;10:1266–73. 31949947 10.21037/jgo.2019.09.04 PMC6954999

[3] Lim TZ Tan KK Endoscopic stenting in colorectal cancer. J Gastrointest Oncol 2019;10:1171–82. 31949937 10.21037/jgo.2019.02.15 PMC6955010

[4] Lee MKC Loree JM Current and emerging biomarkers in metastatic colorectal cancer. Curr Oncol 2019;26: Suppl 1: S7–15. 31819705 10.3747/co.26.5719 PMC6878935

[5] Dekker E Tanis PJ Vleugels JLA Colorectal cancer. Lancet 2019;394:1467–80. 31631858 10.1016/S0140-6736(19)32319-0

[6] Rawla P Sunkara T Barsouk A Epidemiology of colorectal cancer: incidence, mortality, survival, and risk factors. Prz Gastroenterol 2019;14:89–103. 31616522 10.5114/pg.2018.81072 PMC6791134

[7] Ladabaum U Dominitz JA Kahi C Strategies for colorectal cancer screening. Gastroenterology 2020;158:418–32. 31394083 10.1053/j.gastro.2019.06.043

[8] García Sánchez J Colonoscopic polypectomy and long-term prevention of colorectal-cancer deaths. N Engl J Med 2012;366:687–96. 22356322 10.1056/NEJMoa1100370 PMC3322371

[9] Corley Douglas A Jensen Christopher D Marks Amy R Adenoma detection rate and risk of colorectal cancer and death. N Engl J Med 2014;370:1298–306. 24693890 10.1056/NEJMoa1309086 PMC4036494

[10] Jeong MA Kang HW Early-onset colorectal cancer. Korean J Gastroenterol 2019;74:4–10. 31344768 10.4166/kjg.2019.74.1.4

[11] Stark UA Frese T Unverzagt S What is the effectiveness of various invitation methods to a colonoscopy in the early detection and prevention of colorectal cancer? Protocol of a systematic review. Syst Rev 2020;9:49. 32143683 10.1186/s13643-020-01312-x PMC7059336

[12] Goodwin BC Ireland MJ March S Strategies for increasing participation in mail-out colorectal cancer screening programs: a systematic review and meta-analysis. Syst Rev 2019;8:257. 31685010 10.1186/s13643-019-1170-x PMC6827213

[13] Wieszczy P Regula J Kaminski MF Adenoma detection rate and risk of colorectal cancer. Best Pract Res Clin Gastroenterol 2017;31:441–6. 28842054 10.1016/j.bpg.2017.07.002

[14] Zippi M Hong W Crispino P New device to implement the adenoma detection rate. World J Clin Cases 2017;5:258–63. 28798920 10.12998/wjcc.v5.i7.258 PMC5535316

[15] Brand EC Wallace MB Strategies to increase adenoma detection rates. Curr Treat Options Gastroenterol 2017;15:184–212. 28138858 10.1007/s11938-017-0126-2

[16] Rex DK Schoenfeld PS Cohen J Quality indicators for colonoscopy. Gastrointest Endosc 2015;81:31–53. 25480100 10.1016/j.gie.2014.07.058

[17] Kaminski MF Wieszczy P Rupinski M Increased rate of adenoma detection associates with reduced risk of colorectal cancer and death. Gastroenterology 2017;153:98–105. 28428142 10.1053/j.gastro.2017.04.006

[18] Rameshshanker R Tsiamoulos Z Wilson A Endoscopic cuff-assisted colonoscopy versus cap-assisted colonoscopy in adenoma detection: randomized tandem study-DEtection in Tandem Endocuff Cap Trial (DETECT). Gastrointest Endosc 2020;91:894–904. 31836474 10.1016/j.gie.2019.11.046

[19] Sola-Vera J Catalá L Uceda F Cuff-assisted versus cap-assisted colonoscopy for adenoma detection: results of a randomized study. Endoscopy 2019;51:742–9. 31096275 10.1055/a-0901-7306

[20] De Palma GD Giglio MC Bruzzese D Cap cuff-assisted colonoscopy versus standard colonoscopy for adenoma detection: a randomized back-to-back study. Gastrointest Endosc 2018;87:232–40. 28082115 10.1016/j.gie.2016.12.027

[21] Shamseer L Moher D Clarke M PRISMA-P Group Preferred reporting items for systematic review and meta-analysis protocols (PRISMA-P) 2015: elaboration and explanation. BMJ 2015;349:g7647. 10.1136/bmj.g7647 25555855

[22] Whiting PF Rutjes AW Westwood ME QUADAS-2: a revised tool for the quality assessment of diagnostic accuracy studies. Ann Intern Med 2011;155:529–36. 22007046 10.7326/0003-4819-155-8-201110180-00009

[23] Deeks JJ Macaskill P Irwig L The performance of tests of publication bias and other sample size effects in systematic reviews of diagnostic test accuracy was assessed. J Clin Epidemiol 2005;58:882–93. 16085191 10.1016/j.jclinepi.2005.01.016

